# TOWARD AN OPERATIONAL DEFINITION OF COGNITIVE LIFESTYLE: MODELING RESERVE ACROSS THE LIFESPAN

**DOI:** 10.1093/geroni/igz038.1800

**Published:** 2019-11-08

**Authors:** Lisa Swann, Fiona E Matthews

**Affiliations:** Newcastle University, Newcastle upon Tyne, United Kingdom

## Abstract

A recent systematic review of current definitions of cognitive reserve across the lifespan was undertaken by our group and five reserve constructs were identified: Educational attainment, sociological position, occupational complexity, cognitive ability, and engagement in leisure activities. The aim of this study was to test whether the different constructs are predictive of cognitive performance in older adults. A theoretical model of cognitive lifestyle was designed to assess reserve across the lifespan and the different measures were mapped in individuals aged >=65 years (N=7,762; 54.47% women) from the Cognitive Function and Ageing Study (CFAS II). Multivariable logistic regression analyses, controlling for age and sex, were used to determine the relationship between later life cognitive function and the five identified measures of reserve. In support of previous findings, the results show that risk of cognitive decline decreases with additional education (OR=0.89; 95% CI= 0.86, 0.94), increasing fluid intelligence (OR=1.00; 95% CI= 0.99, 1.00), and participation in leisure activities (OR= 0.27; 95% CI=0.17, 0.42), and that the lower the hierarchical position of a person’s social strata, the greater their odds of cognitive impairment in later life (OR=1.02; 95% CI=1.00, 1.05). However, though greater occupational complexity with data (OR=1.11; 95% CI=1.03, 1.19) and with people (OR=1.10; 95% CI= 1.03, 1.19) was found to be protective of cognitive decline as expected, greater occupational complexity with things was associated with greater decline (OR=0.98; 95% CI=0.93, 1.04). Given the cross-sectional nature of the present study, further longitudinal work is needed to ascertain what is driving these results.

